# Effects of elevated nutrient supply on litter decomposition are robust to impacts of mammalian herbivores across diverse grasslands

**DOI:** 10.1007/s00442-025-05791-4

**Published:** 2025-09-13

**Authors:** Adrienne B. Keller, Elizabeth T. Borer, Christopher R. Buyarski, Elsa E. Cleland, Allison Gill, Andrew S. MacDougall, Joslin L. Moore, John W. Morgan, Rebecca L. McCulley, Anita C. Risch, Eric W. Seabloom, Justin Wright, Sarah E. Hobbie

**Affiliations:** 1 Department of Ecology, Evolution and Behavior, University of Minnesota, St. Paul, MN 55108 USA; 2 College of Forest Resources and Environmental Science, Michigan Technological University, Houghton, MI, USA; 3 HIT Lab NZ, University of Canterbury, Christchurch, New Zealand; 4 Ecology, Behavior & Evolution Department, University of California San Diego, La Jolla, San Diego, CA 92093 USA; 5 Department of Biology, Williams College, Williamstown, MA 01267 USA; 6 Department of Integrative Biology, University of Guelph, Guelph, ON N1G2W1 Canada; 7 Department of Energy, Environment and Climate Action, Arthur Rylah Institute for Environment Research, Heidelberg, VIC 3084 Australia; 8 School of Biological Sciences, Monash University, Clayton, VIC 3084 Australia; 9 School of Agriculture, Food and Ecosystem Sciences, The University of Melbourne, Melbourne, VIC 3010 Australia; 10 Department of Environment and Genetics, La Trobe University, Bundoora, VIC 3010 Australia; 11 Department of Plant and Soil Sciences, University of Kentucky, Lexington, KY 40546-0312 USA; 12 Swiss Federal Institute for Forest, Snow and Landscape Research WSL, 8903 Birmensdorf, Switzerland; 13 Department of Biology, Duke University, Durham, NC 27708 USA

**Keywords:** Grasslands, Herbivory, Litter decomposition, Nutrient addition, Nutrient network (NutNet)

## Abstract

Litter decomposition is one of the largest carbon (C) fluxes in terrestrial ecosystems and links aboveground biomass to soil C pools. In grasslands, decomposition drivers have received substantial attention but the role of grassland herbivores in influencing decay rates is often ignored despite their potentially large effects on standing biomass and nutrient cycling. Recent work has demonstrated that nutrient addition increases early-stage decay and suppresses late-stage decay. Mammalian herbivores can mediate the effects of nutrient supply on biomass, suggesting herbivores may alter the effects of nutrients on decomposition, though this is largely unknown. We examined how herbivory mediates the effects of nutrient supply on long-term decomposition across 19 grassland sites of the Nutrient Network distributed experiment. At each site, a full-factorial experiment of combined nitrogen (N), phosphorus (P), and micronutrient (K) enrichment (‘control’ or ‘ + NPK’) and mammalian herbivore (> ~ 50 g) exclusion (‘unfenced’ or ‘fenced’) was carried out in a randomized block design. We hypothesized that nutrient effects on litter decomposition would be strongest where herbivores caused the greatest reductions in aboveground plant biomass (i.e., at sites with more intense herbivory). After accounting for wide variation in decomposition rates across sites, we found that, within sites, elevated nutrients increased early-stage decay and suppressed late-stage decay. In contrast, neither herbivore exclusion (i.e., fencing) nor site level changes in aboveground biomass due to herbivory altered the nutrient effects on decomposition rates. Across grasslands, our results indicate that elevated nutrient supply modifies litter decomposition rates independent of herbivore impacts.

**Supplementary Information:**

The online version contains supplementary material available at 10.1007/s00442-025-05791-4.

## Introduction

Decomposition of dead plant material is one of the largest carbon (C) fluxes in terrestrial ecosystems, influencing how much C is sequestered in the soil versus returned to the atmosphere as CO_2_ (Swift et al. 1979). Consequently, understanding how litter decomposition responds to environmental change is critical for accurately predicting global C cycling and climate-carbon feedbacks. Determining the effect of global change on litter decomposition is particularly pertinent in grasslands, which cover ~ 25% of the terrestrial land globally and are experiencing some of the strongest effects of environmental change (Asner et al. 2004; Blair et al. 2014). Specifically, aboveground herbivory by mammalian herbivores (hereafter, ‘herbivores’, including both wild herbivores and domesticated livestock)—a primary factor shaping grassland ecosystems—is being dramatically altered in many regions due to anthropogenic activities, such as hunting, livestock husbandry, and habitat conversion (Blair et al. 2014; Ripple et al. 2015). Simultaneously, many grasslands are experiencing elevated nutrient loads due to increased atmospheric N deposition and agricultural nutrient runoff (Wang and Li 2019; Ackerman et al. 2019). Herbivory and nutrient supply rates may have interactive effects on C cycling, in particular via consumer activity effects on plant productivity and biomass that can depend on nutrient supply (Borer et al. 2020). The divergent spatial patterns of live plant biomass and litter loss across regions and continents suggest that factors such as climate, nutrient supply, and herbivory likely have differing, and potentially interacting, effects on litter decomposition (O’Halloran et al. 2013). However, potential interactive effects of herbivory and nutrient supply on the process of litter decomposition remain poorly understood.

**Fig. 1:**
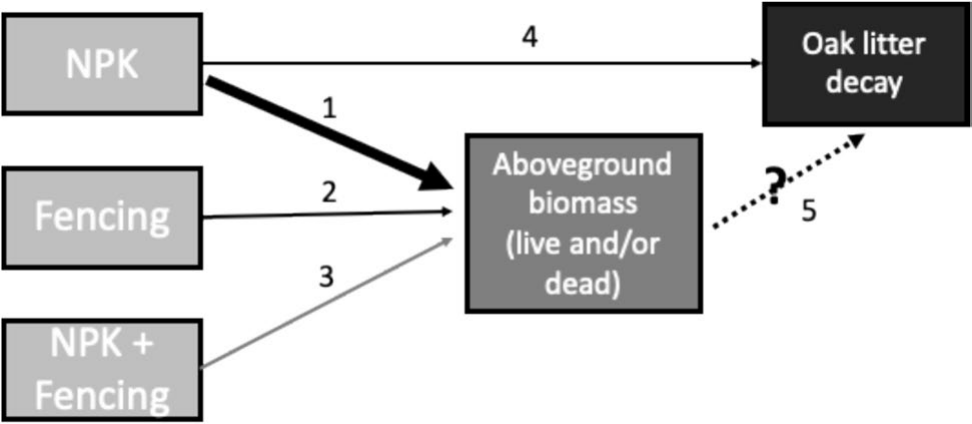
Conceptual figure of pathways by which nutrient supply (NPK) and herbivore exclusion (fencing) may influence litter decomposition (of a common novel oak leaf litter) within a site. These include direct and indirect impacts via aboveground plant biomass (live and/or dead). Our work builds on prior research that has investigated individual pathways (indicated by solid arrows and associated numbers and described below). Treatment variables are shown in light grey boxes, mediating covariates are shown in darker grey, and the response variable is shown in a black box. The two thickness levels of solid arrows qualitatively represent the relative magnitude of the treatment effect compared to other pathways included in the figure. Black solid arrows indicate a significant effect, and the grey solid arrow indicates no significant effect as shown in published literature

While elevated nutrient inputs in grasslands are known to affect both plant biomass (Fay et al. 2015) and decomposition rates independent of variation in plant chemistry (Gill et al. 2022), field experimental tests of these nutrient impacts on grassland C cycling have generally been carried out in the presence of herbivores. This makes it difficult to assess whether nutrients are directly affecting decomposition or if the observed effects are mediated by herbivore responses to increased nutrient supply. For example, herbivores remove more grassland plant biomass with increasing nutrient supply rates (Borer et al. 2020), with potential downstream effects on decomposition rates given the influence of plant biomass on the local decomposition environment. This suggests that herbivore effects will interact with nutrient supply to influence litter decomposition patterns via effects on aboveground biomass, notwithstanding plant community compositional changes (Fig. 1). In addition, in the presence of herbivores, elevated N (alone or with other macronutrients) has been shown to stimulate early-stage decomposition but suppress late-stage decomposition of a common substrate across grasslands (Gill et al. 2022). This temporal change in the effect of nutrient supply on decomposition in the presence of herbivores points to the hypothesis that the effects of herbivory may underlie this temporal variation.

At local scales, aboveground biomass-mediated effects of herbivore activity and elevated nutrients on litter decomposition may occur through multiple mechanisms. Aboveground biomass, controlled in part by herbivores (Borer et al. 2020), can alter litter exposure to solar radiation and, consequently, moisture at the soil–litter interface. On one hand, increased penetration of solar radiation due to reduced biomass pools may increase litter decomposition given that photodegradation is a key driver of litter decomposition in many grasslands (King et al. 2012). On the other hand, increased solar radiation may reduce litter layer moisture, thereby suppressing litter decomposition, particularly in more arid sites, where water availability can strongly constrain decomposition (Gholz et al. 2000). Other effects of aboveground biomass on the decomposition environment may include changes in soil temperature due to shading and evaporative cooling, freeze–thaw events, and protection from soil erosion (King et al. 2012).

Herbivore effects on aboveground biomass can vary among sites, because differing abundance and identity of herbivores cause differing site-level herbivore interactions with plants (Chang et al. 2018, Hao and He 2019, Borer et al. 2020). Any effects of herbivores on decomposition that appear mediated by effects on plant biomass might also arise because of non-consumptive effects of herbivores (e.g., trampling, nutrient inputs through waste). Combined, these consumptive and non-consumptive effects on decomposition should be greater in sites where the herbivore community has larger net effects on plant biomass. Thus, while herbivore effects on litter decomposition patterns are likely mediated by (or associated with) plot-scale changes in live and dead aboveground biomass, these changes occur on a backdrop of site variation in herbivore intensity (Anderson et al. 2018), highlighting the importance of studying sites spanning a range of intensities of herbivore impacts.

Site factors also may modulate how herbivory and its interaction with nutrient supply influence litter decomposition. Climate can alter both aboveground plant biomass and decomposition rates (Swift et al. 1979; Blair et al. 2014). Across grasslands globally, herbivore exclusion increases aboveground biomass under fertilized conditions at sites with low precipitation, highlighting an important herbivore by nutrient supply interaction that also depends on site conditions (Borer et al. 2020). For domestic livestock, cattle grazing increased litter extracellular enzyme activities and decomposition in mesic, but not dry, temperate grasslands (Chuan et al. 2018, 2020). Nitrogen fertilization also may amplify positive effects of atmospheric N deposition on litter decomposition rates across grasslands (Gill et al. 2022). It remains untested how these site factors may interact with herbivore effects, with or without fertilization, to drive differences in litter decomposition across sites.

Here, we examined how herbivory, with and without nutrient enrichment, influenced litter decomposition of a standard leaf litter substrate (*Quercus ellipsoidalis*). We then assessed if such herbivore and nutrient supply effects on litter decomposition were mediated by changes in aboveground biomass and how other plot- and site-level characteristics affected this relationship. Acknowledging that herbivore identity, abundance, diet, and habits (e.g., trampling) varied across sites, we aimed to assess the influence of herbivory (with varying characteristics and intensities across sites) on plant biomass and litter decomposition. We conducted this experiment across 19 grassland sites that are part of the Nutrient Network (NutNet; www.nutnet.org), a coordinated research network focused on understanding how nutrient supply (via fertilization) and herbivory (via fencing to exclude mammalian herbivores) affect grassland dynamics worldwide (Borer et al. 2017). As shown in Fig. 1, Borer et al. (2020) found that across 58 NutNet grassland sites (1) NPK increased live aboveground plant biomass by 58% on average, (2) fencing increased live aboveground biomass by 12% on average, and (3) there was no significant interactive effect of NPK and fencing on live aboveground biomass. In addition, (4) Gill et al. (2022) and Ochoa‐Hueso et al. (2020) found that NPK slightly increased early stage litter decomposition of a novel litter substrate compared to control plots across 20 and 21 NutNet grassland sites, respectively. Finally, (5) this study tied these individual studies together to examine the degree to which live and dead aboveground biomass mediates treatment effects on litter decomposition, with the expectation that NPK effects on litter mass remaining would be mediated by aboveground biomass, while the effects of fencing (alone and in combination with NPK) on litter decay would be minimal given weak fencing effects on biomass. We further tested whether these within-site relationships were contingent on site-level biotic and abiotic conditions.

## Methods

### Study sites and decomposition experimental design

This study included 19 NutNet grassland sites spanning Australia, Europe, and North America. Climatic conditions varied substantially across sites, with mean annual temperature (MAT) ranging from 0 to 18ºC, mean annual precipitation (MAP) ranging from 246 to 1877 mm, and moisture index (MAP/potential evapotranspiration) ranging from 0.26 to 2.44. Sites also varied in total atmospheric N deposition (1.8–18.9 kg N ha^−1^ yr^−1^) and grassland type (Table 1). Such site variation allowed for statistical examination of how site conditions in combination with experimental manipulation of nutrient supply and herbivory affected litter decomposition.

**Table 1:** Characteristics of sites included in this study, listed in order by grassland type and then MAT

Site code	Country	Latitude (°)	Longitude (°)	Grassland type	Elevation (m)	MAP (mm)	MAT (°C)	N Dep (kg N/ha/yr)
valm.ch	CH	46.63	10.37	alpine grassland	2320	681	0.1	18.9
bogong.au	AU	-36.87	147.25	alpine grassland	1760	1678	6.0	5.1
hopl.us	US	39.01	− 123.06	annual grassland	598	1065	13.2	3.4
mcla.us	US	38.86	− 122.41	annual grassland	642	936	14.0	3.4
sier.us	US	39.24	− 121.28	annual grassland	197	936	16.3	3.4
elliot.us	US	32.88	− 117.05	annual grassland	200	344	17.7	6.6
sage.us	US	39.43	− 120.24	montane grassland	1920	831	5.8	3.4
bnch.us	US	44.28	− 121.97	montane grassland	1318	1618	6.8	2.8
look.us	US	44.21	− 122.13	montane grassland	1500	1877	6.9	2.8
cowi.ca	CA	48.46	-123.38	old field	50	762	10.4	3.7
unc.us	US	36.01	− 79.02	old field	141	1157	14.9	13.1
spin.us	US	38.14	− 84.50	pasture	271	1152	12.5	13.9
hall.us	US	36.87	− 86.70	restored tallgrass prairie	194	1289	13.8	14.3
kiny.au	AU	-36.20	143.75	semiarid grassland	90	408	15.6	2.1
burrawan.au	AU	-27.73	151.14	semiarid grassland	425	643	18.2	2.3
bldr.us	US	39.97	-105.23	shortgrass prairie	1633	487	9.9	1.9
shps.us	US	44.24	-112.20	shrub steppe	910	246	5.3	1.8
cdcr.us	US	45.43	-93.21	tallgrass prairie	270	740	6.3	7.0
cbgb.us	US	41.79	-93.39	tallgrass prairie	275	871	9.3	18.0

At each site, we carried out a full factorial nutrient (nitrogen (N), phosphorus (P), and potassium plus micronutrients (K), ‘ + NPK’, or ‘control’) and herbivore exclusion (‘unfenced’ or ‘fenced’) experiment. These treatments were initiated < 1–2 years prior to when the decomposition experiment was deployed, depending on the site. Prior work from this experiment has shown that the direction and magnitude of the effect of fences on plant biomass are similar across years (Borer et al. 2020). The experiment was conducted in a replicated block design using 5 × 5 m plots for all treatments, with plots separated by at least 1 m in all cases. All sites included 3 blocks except for Sierra Foothills (sier.us) which had 5 blocks and Boulder South Campus (bldr.us) which had 2 blocks. NPK-treated plots received 10 g N m^−2^ yr^−1^ as timed-release urea, (NH_2_)_2_CO, 10 g P m^−2^ yr^−1^ as triple-super phosphate Ca(H_2_PO_4_)_2_, and 10 g K m^−2^ yr^−1^ as potassium sulphate K_2_SO_4_ annually. A one-time addition of micronutrient mix (Fe (15%), S (14%), Mg (1.5%), Mn (2.5%), Cu (1%), Zn (1%), B (0.2%) and Mo (0.05%)) was also applied at the start of the experiment to all nutrient addition plots. Herbivore-exclusion plots were fenced to exclude aboveground mammalian herbivores (> ~ 50 g). Fences were 2.30 m tall and included a 0.30-m outward-facing flange stapled to the ground to exclude some digging animals, but subterranean mammals were not excluded. For those few species capable of jumping > 2 m, the height of the fences and relatively small size of the plots strongly discouraged individuals from choosing to enter experimental plots. Some sites deviated slightly from this fence design; for details, see SI Table 3. Herbivore identity varied across sites and represented herbivore lineages that have arisen through evolutionary history in the different regions of this study. Only two sites had domestic herbivores present at low intensities (burrawan.au had cattle present; shps.us had sheep present).

In a complementary experiment at the same sites, Gill and others (2022) examined the individual and combined effects of N, P, and K addition on decomposition dynamics. Note that one site (Sedgwick; sedg.us) included in the Gill and others (2022) study did not include the fencing treatment and, therefore, is not included here. Here, we expand on their analysis to explore how herbivore exclusion, alone and in combination with nutrient addition, affects early and late-stage litter decomposition. For full details on the decomposition experimental design and sample analysis, see Gill et al. (2022). Briefly, *Quercus ellipsoidalis* leaf litter was selected as the decomposing substrate, because it was readily available, had a N concentration similar to grassland litter (Wedin and Pastor 1993), and was novel to all sites, thereby eliminating the potential for home-field advantage effects at any site (Gholz et al. 2000; Ayres et al. 2009; Yuan et al. 2019). Leaf litter was collected from several adjacent trees at the Cedar Creek Ecosystem Science Reserve in Minnesota, USA. Litter bags (20 cm × 20 cm) were constructed of 1-mm mesh fiberglass window screen and were filled with 10 g (dry weight) of sterilized (autoclave at 121 °C for 15 min) leaf litter. Bags were strung together in groups of seven and pinned to the ground (1 string per plot) and deployed between December 2009 and October 2010 depending on the site. The duration of the decomposition experiment varied from site to site. One bag per year was harvested at approximately annual intervals for up to 7 years. The total number of sites included for a given year in the final data set varied, ranging from three to seven harvests per site. To account for any soil contamination, proportion initial mass remaining was converted to proportion initial carbon (C) remaining as described in Gill et al. (2022). Hereafter, “litter mass” is used to refer to values converted to litter C.

### Decomposition models

Gill and others (2022) examined four alternative statistical models of litter decomposition. In their study, the single and asymptotic exponential models and the Weibull model best described the litter decomposition trajectory, while the double exponential model was never the best model. Therefore, we fit our data to these three models*.* Briefly, the single exponential decay model considers a single pool of litter with a constant decay rate (*k*_*s*_) in the form: ${X=e}^{{-k}_{s}t}$. The asymptotic exponential model considers two litter pools with different but constant decomposition rates: a slow fraction (*A*) with decomposition rate of zero and a labile fraction (1 – *A*) with decomposition rate *k*_⍺_. The asymptotic exponential model takes the form $X=A+\left(1-A\right){e}^{{-k}_{\alpha }t}$. In contrast to single and asymptotic exponential models, the Weibull model describes litter decay as a continuous distribution of residence times. The Weibull model takes the form $X={e}^{-({\frac{t}{\beta })}^{\alpha }}$, where β and ⍺ are scale and shape parameters, respectively, that describe how litter decay changes through time. The Weibull model’s continuous Change in decomposition rate cannot be directly compared to decomposition rates of discrete litter pools as described by the other two models. Instead, we describe early stage litter decay by calculating time to 10%, 25%, and 50% mass loss (*t*_*1/10*_, *t*_*1/4*_, and *t*_*1/2*_, respectively) and overall decay rate by calculating mean residence time (*MRT*). For full details of the models, see Gill and others (2022).

### Covariate measurements

To understand variation in decomposition patterns within and across sites, we examined several plot- and site-level factors related to environmental conditions, herbivory, and vegetation biomass. Specifically, mean annual temperature (MAT), mean annual precipitation (MAP), precipitation seasonality (precipitation in the wettest month/MAP), and moisture index (MAP/potential evapotranspiration) were extracted at 1 km spatial resolution for each site from the CIGAR–CSI/BIOCLIM database spanning 1970–2000 (Hijmans et al. 2005). Modeled atmospheric N deposition was determined for each site from Ackerman and others (2019). Due to the spatial resolution of this data product (2º–2.5º), some sites are assigned the same N deposition value. Site-level herbivory was estimated by comparing the Change in aboveground biomass between year 1 and year 0 of the experiment in unfenced (control) versus fenced plots. Specifically, herbivore intensity was calculated as

1 $$ln\frac{\frac{AG biomass in control plots year 1}{AG biomass in control plots year 0}}{\frac{AG biomass in fenced plots year 1}{AG biomass in fenced plots year 0}}$$

A positive log response ratio for herbivore intensity indicates herbivory increased aboveground biomass, while a negative log response ratio indicates herbivory decreased aboveground biomass. In a 1 m^2^ subplot in each plot, the % cover of each plant species was estimated visually and summed to total % cover per plot. Photosynthetically active radiation (PAR) was measured at ground-level (PAR_Ground_) and above the grassland canopy (PAR_Ambient_) using a linear ceptometer. Proportion of PAR at ground level (proportion PAR) was calculated as PAR_Ground_/PAR_Ambient_. Total aboveground biomass was measured by clipping all biomass in two 0.1 m^2^ strips, sorting live (current year’s growth) and dead (previous years’ growth) biomass, drying to constant mass at 60º C, and weighing to the nearest 0.01 g. Cover, PAR, and aboveground biomass were measured at peak biomass in most years. Given that the number of years that vegetation was sampled and the duration of the decomposition experiment varied among sites in the study, vegetation measurements were averaged over all years with available data for the time frame of the study for each site.

### Data analysis

To examine how herbivory, alone and in interaction with nutrient supply, affects litter decomposition dynamics, linear mixed models were fit to each decomposition parameter (*lme* function; *nlme v. 3.1.164* R package). NPK and fencing and the interaction between the two were included as fixed effects, and site was included as a random intercept (model form: decomposition parameter ~ NPK + Fencing + NPK: Fencing, random = ~ 1 | site). Sample size did not allow inclusion of site as a random slope in the mixed models. Block accounted for very little variation in any model and, therefore, was not included as a nested random effect. Subsequent analyses were carried out only for the decomposition parameters, wherein at least one treatment was significant in the treatment-only model (⍺ < 0.05), i.e., early stage Weibull *t*_*1/10*_ and late-stage asymptotic *A*.

Piecewise structural equation models (SEM), which allow for nested experimental designs and random effects, were used to explicitly test how aboveground live and dead biomass mediate treatment effects on litter decomposition (*piecewiseSEM v. 2.1* R package; Lefcheck 2016). Piecewise SEMs were constructed for Weibull *t*_*1/10*_ and late-stage asymptotic *A*, respectively, using the same initial model that included two components: one predicting aboveground biomass (referring to arrows 1–3 in Fig. 1) and the second predicting the decomposition parameter (referring to arrow 5 in Fig. 1). Linear mixed models were fit for each component, with site treated as a random intercept in all cases. For each model, the *stepAIC()* function in the *MASS v. 7.3.60.2* R package (Ripley 2024) was used to determine if a composite variable composed of a linear combination of live and dead aboveground biomass was a better predictor of the decomposition parameter than live or dead aboveground biomass alone. A square root transformation was applied to aboveground live and dead biomass to improve normality of the residuals. Given that one site lacked dead aboveground biomass (shps.us), this step was performed using a subset of the data set that excluded shps.us. However, in both models, live aboveground biomass was a better predictor and was used rather than a composite variable, and therefore, final model analysis was performed on the full data set. Overall model fits of the piecewise SEMs were evaluated using Fisher’s C statistic. Significant pathways were identified using an ⍺ < 0.05 significant level cutoff.

Finally, to examine how other plot- and site-level factors may explain site variation in the effects of herbivory and nutrient supply on litter decomposition, linear mixed models were fit with the same random effect structure as in the treatment-only models, but covariates were included along with treatments as fixed factors. We focused on factors with the potential to strongly affect litter layer dynamics, and, therefore, did not examine edaphic factors, such as soil nutrients or pH. Initial covariates included moisture index, precipitation seasonality, atmospheric N deposition, herbivory intensity, % cover, proportion PAR, and live aboveground biomass (for the same reasons as in the SEM analysis described above, dead aboveground biomass was not included). Prior to model fitting, each predictor variable was assessed for normality. A square root transformation was applied to aboveground live and % cover was log transformed to improve normality. Model averaging was used to determine which covariates had significant effects on litter decomposition patterns using the *MuMIn() v.1.48.4* R package (Bartoń 2023). Likelihood ratio tests showed that a model without interactions between covariates and treatments was not significantly different from a model with first-order interactions between covariates and treatments (Weibull *t*_*1/10*_: *P* = 0.48; asymptotic *A*: *P* = 0.97); therefore, covariate × treatment interactions were not included in model averaging analyses. Standardized models were used, with treatments included in all possible models. Model averages were assessed using all models (i.e., full averaging) within 4 AIC units of the best model. All data analysis was performed in R (R version 4.3.2, R Foundation for Statistical Computing 2013).

## Results

### Nutrient supply, but not herbivore exclusion, affected within-site rates of litter decomposition

**Fig. 2:**
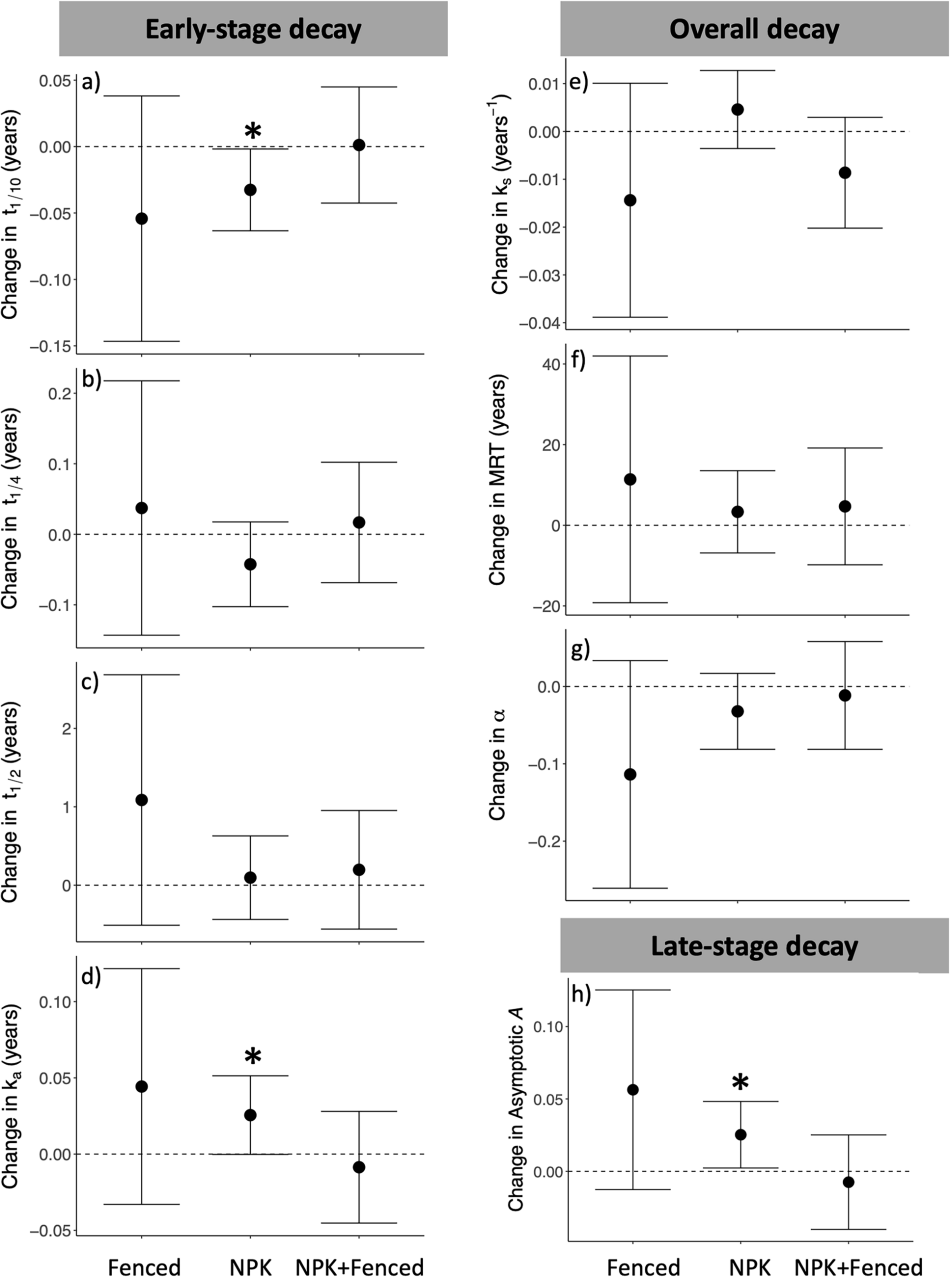
Effects of fencing and nutrient addition on average within-site litter decomposition parameters. Parameters describing early stage decay include **a** Weibull *t*_*1/10,*_ **b** Weibull *t*_*1/4,*_ **c** Weibull *t*_*1/2,*_ and **d** *k*_*⍺*_; overall decay parameters include **e** *k*_*s,*_ **f** *MRT,* and **g** *⍺*; and late-stage decay is described by Asymptotic *A*. Plotted values are the differences of decomposition (as calculated from litter C remaining) in the treatment compared to the control plots, estimated using mixed-effects models with no covariates. Error bars are standard error of the slope estimates. Slopes that are significantly different from zero (*P* ≤ 0.05) are indicated with *. An interaction of zero (NPK + Fenced) indicates additive effects of NPK and Fenced effects. A decrease in any of the Weibull *t*_*1/x*_ parameters or an increase in *k*_*⍺*_ indicates accelerated early stage decay. An increase in Asymptotic *A* indicates slowed late-stage decay

Litter decay parameters varied widely among sites (SI Table 1). After accounting for among site variation, we found that nutrient addition increased early-stage decay of the standard oak litter substrate (increased *k*_*⍺*_: *P* = 0.05; decreased Weibull *t*_*1/10*_: *P* = 0.04) and suppressed late-stage decay (increased asymptotic *A*: *P* = 0.03). Herbivore exclusion did not significantly affect litter decay at any stage. There were also no significant interactive effects between nutrient addition and herbivore exclusion on decay (SI Table 2, Fig. 2). The range of the effect of herbivore exclusion was greater than the range of nutrient addition effects for all decomposition parameters.

**Table 2:**
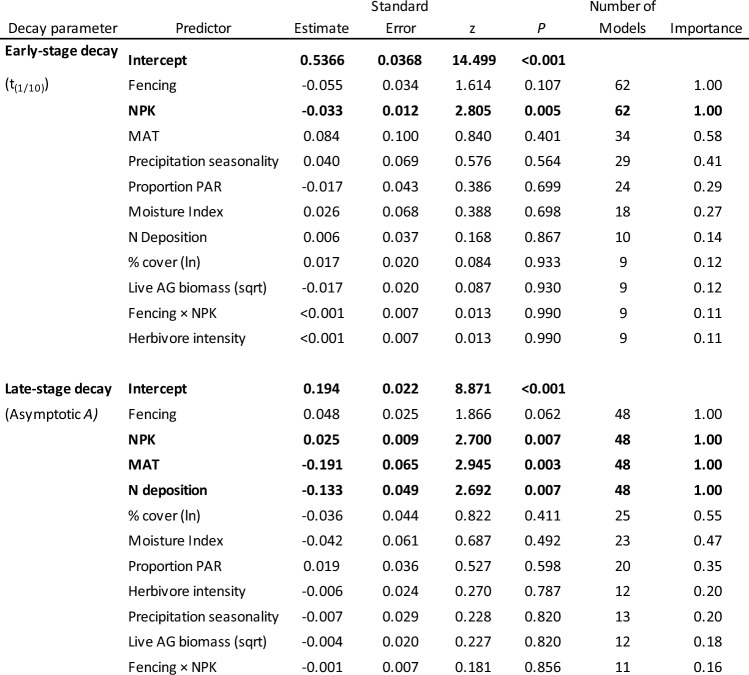
Effects of experimental treatments and local conditions on early- and late-stage litter decomposition .The results stem from multi-model inferences using a suite of mixed effects models with full averaging applied. The parameter’s importance is determined by the cumulative Akaike weights across models that fall within 4 AICc units of the top model (the model with the lowest AICc) that include the parameter. Importance values span from 0 (indicating no explanatory contribution) to 1 (indicating presence in all top models). All tests are two-tailed. Predictors that are bold are statistically significant at *P* < 0.05

### No evidence of aboveground biomass-mediated effects of treatments on litter decomposition

**Fig. 3:**
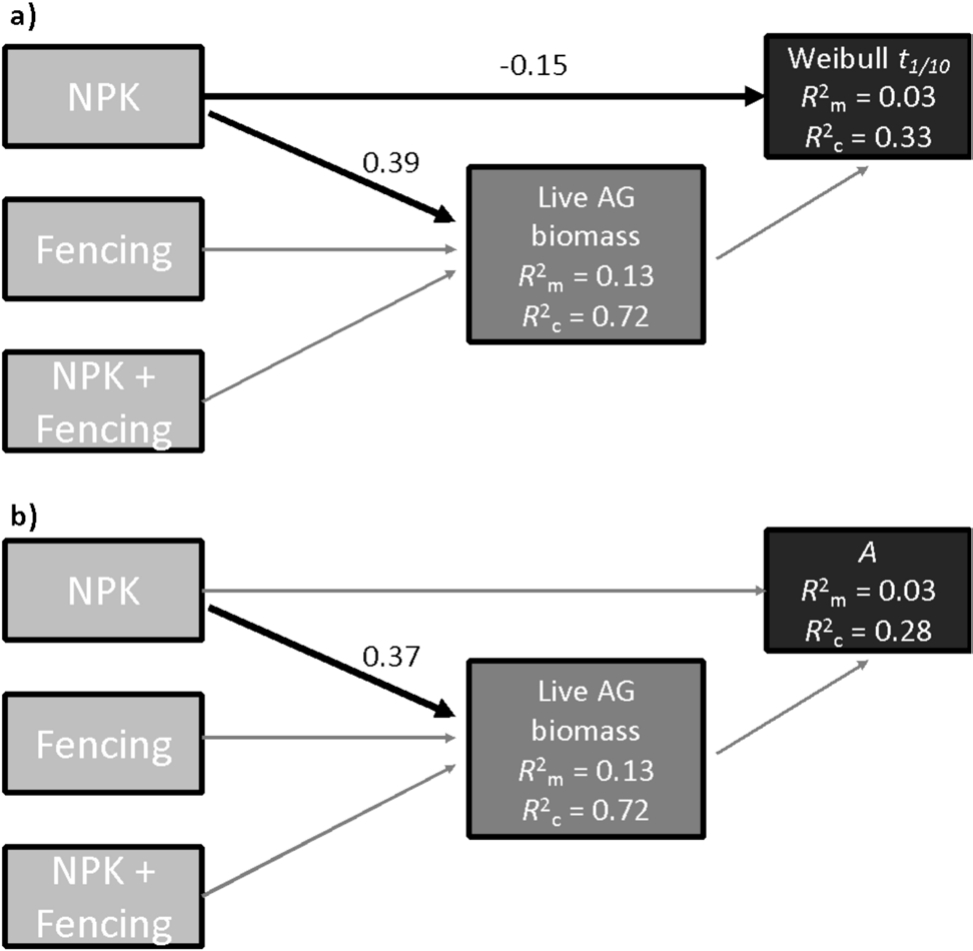
Structural equation models evaluating aboveground biomass-mediated effects of treatments on early-stage **a** and late-stage **b** litter decomposition parameters. Black arrows indicate significant pathways and the standardized coefficients for these pathways are presented beside the corresponding arrow. Grey arrows indicate not-statistically significant pathways. Marginal *R*^2^ (*R*^2^_m_) values and conditional *R*^2^ (*R*^2^_c_) values are shown for each component of the piecewise SEM, indicating the variability explained by the fixed effects or the fixed and random effects, respectively

NPK increased live aboveground plant biomass (*P* < 0.001; *R*^2^_marginal_ = 0.13, *R*^2^_conditional_ = 0.72), while herbivore exclusion alone did not significantly affect live aboveground biomass at these study sites (SI Fig. 1), and there was no significant interaction between NPK and herbivore exclusion. Dead aboveground biomass was not significantly affected by the treatments (SI Fig. 1). Using piecewise SEM, we did not find evidence for aboveground biomass-mediated effects of nutrient supply or herbivore exclusion on litter decay patterns. In models of both early- (Weibull *t*_*1/10*_) and late- (*A*) stage decay, NPK significantly stimulated aboveground biomass. In the SEM, there was also a significant direct effect of NPK on early-stage, but not late-stage, decay. The pathway between biomass and litter decay was not supported in either SEM (Fig. 3). Within-site effects of treatments on decomposition (i.e., *R*^2^_marginal_) occurred on a backdrop of substantial among-site variation in decomposition (i.e., *R*^2^_conditional _– *R*^2^_marginal_), pointing to the potential for an influential role of site-level biotic and abiotic conditions.

### Site-level conditions: MAT and N deposition stimulated late-stage decay

In addition to aboveground biomass, we expected other local conditions to explain site variation in treatment effects on litter decay patterns. None of our hypothesized covariates were significant predictors of early-stage decay rates. In contrast, both MAT and N deposition were significant covariates in models predicting late-stage decay (asymptotic *A*: MAT, *P* = 0.003; N deposition,* P* = 0.007), with sites that were warmer and/or experienced greater N deposition exhibiting faster late-stage decay, as indicated by a smaller fraction of slowly decomposing litter, compared to sites that were cooler and/or had lower N deposition (Table 2).

## Discussion

Across multiple continents the exclusion of aboveground mammalian herbivores did not have consistent effects on litter decomposition in grasslands, supporting our hypothesis. Increased nutrient supply did stimulate early stage decomposition and suppressed late-stage decomposition as shown previously (Gill et al. 2022). Contrary to our expectation based on factors controlling both plant biomass and soil C, herbivores did not mediate the effect of nutrient supply on decomposition of a novel substrate. In addition, the effects of nutrients and herbivore exclusion on aboveground biomass did not mediate changes in litter decay.

This study showed no consistent effect of fencing on decomposition across sites. While nutrient addition increased aboveground biomass in the presence and absence of herbivores across our study sites, this increase did not significantly modulate the trajectory of litter decomposition according to structural equation modeling. The absence of biomass-mediated effects due to nutrient addition may have arisen if variation in aboveground biomass at these sites did not reduce ground surface insolation sufficiently to alter photodegradation or moisture in the litter layer. Alternatively, an increase in biomass could have decreased insolation while concomitantly increasing moisture, such that the two mechanisms counteracted one another and resulted in a null net effect on decomposition. Moreover, prior research showed that across grasslands globally, live plant biomass and litter disappearance did not covary (O’Halloran et al. 2013). Instead, each showed considerable variation but at different spatial scales: live plant biomass varied across continents, sites, and plots, while litter decomposition primarily varied across regions and continents. This disconnection between plant mass accumulation and loss aligns with our results showing substantial spatial variation but no consistent aboveground biomass-mediated effect on litter decomposition.

The variation in herbivore intensity among sites aligns with prior work showing variable effects on aboveground biomass (Gruner et al. 2008; Borer et al. 2020) but did not help to explain cross-site variation in decomposition patterns, in contrast to our expectations (SI Fig. 2). However, our metric of herbivore intensity was calculated from site-level biomass consumption. The chemical composition of litter inputs could have shifted, as demonstrated by Anderson et al. (2018) for some of our sites, without changes in biomass inputs due to site-specific differences in herbivore intensity. Such shifts could affect nutrient supply and microbial dynamics in the litter layer and thereby explain some of the cross-site variation in decomposition patterns. However, using a novel litter substrate to measure decomposition rates in this study, we isolated the effects of herbivores on the decomposition process. This study design avoided confounding feedbacks that can manifest given that herbivory can alter plant community composition and thus litter chemistry, with effects that vary across sites (Augustine and McNaughton 1998).

In addition, non-trophic effects of herbivore presence, density, and identity likely contribute to variation in decomposition rates across sites (Haynes et al. 2014; Wang et al. 2018). Such non-trophic effects include disturbance (e.g., soil compaction, bioturbation) and resource inputs (e.g., dung and urine deposition), and are increasingly understood to rival trophic (e.g., biomass-mediated) effects (Bardgett and Wardle 2003; Andriuzzi and Wall 2017; Kristensen et al. 2022). Accordingly, in Argentinian cattle-grazed grasslands, herbivore-induced shifts in plant community composition affected decomposition patterns more via changes to the soil environment than to resource inputs (Vaieretti et al. 2013, 2018). In this study, we were not able to separate trophic (biomass-mediated) from non-trophic effects. However, given the lack of consistent fencing effects on decomposition across sites, non-trophic effects likely varied across sites depending on type and intensity of herbivory. Site variation of herbivore effects on decomposition is expected given that herbivore fences act on each site-level herbivore community (Andriuzzi and Wall 2017). Consequently, to illuminate generalizable patterns of herbivore effects on decomposition and C cycling across grasslands, future research will need to consider additional characteristics of the herbivore community. Controlled manipulation of herbivore offtake (e.g., through regular clippings) could advance mechanistic understanding of aboveground biomass-mediated effects on decomposition. Our results also point to the need for direct tests of the relative importance of trophic versus non-trophic effects of herbivory on ecosystem C cycling across grasslands.

Cross-site variation in early stage decomposition of a novel substrate was not explained by any of our measured covariates, while the late-stage decomposition rate increased with both N deposition and MAT across grassland sites. Similar covariate patterns were shown in a companion study to this one that did not consider herbivore impacts on decomposition (Gill et al. 2022). As discussed therein, the positive relationship of late-stage decomposition with MAT likely occurred because of higher temperatures stimulated microbial activity. The positive relationship of late-stage decomposition with N deposition is more puzzling as it contrasts the negative effects of NPK addition. Perhaps the negative effects of fertilization on late-stage decomposition resulted from the high rates of NPK addition, whereas lower rates of N deposition had a stimulatory effect on decomposition rates. Combined with our lack of evidence of interactive effects between herbivore exclusion and covariates in decomposition, these complementary studies suggest that local abiotic environmental factors may qualitatively affect decomposition patterns similarly regardless of herbivore presence.

## Conclusion

Nutrient addition had more consistent effects on grassland ecosystem C cycling across these study sites compared to effects of herbivore exclusion, which were highly variable in magnitude and direction. While elevated nutrient supply and large herbivore exclusion stimulate aboveground biomass in many grassland sites globally (Borer et al. 2020), changes in aboveground biomass do not appear to directly affect rates of litter decomposition. Our work contributes to a growing understanding of how herbivory, alone and in combination with external nutrient supply, affects ecosystem C cycling. Prior work across many of the same grassland sites found that herbivore exclusion dampened the stimulatory effect of nutrient fertilization on soil C pools (Sitters et al. 2020); here, we found no interactive effect of nutrient supply and herbivory on aboveground litter decomposition rates. These contrasting effects on litter decomposition and soil C suggest that factors other than rates of aboveground litter decomposition are driving previously observed interactive effects of herbivore exclusion and nutrient addition on soil C pools. Our results also highlight how variation in the herbivore community among sites can drive differences in litter decomposition rates, highlighting the need for ecosystem C cycling research to explicitly consider the non-trophic effects of herbivores.

## Supplementary Information

Below is the link to the electronic supplementary material.

Supplementary file1 (DOCX 2071 KB)

## Data Availability

Data associated with this study are available here: 10.6073/pasta/39255d27cdf5d950b4810842e33546f1. Data from an associated study (Gill et al. 2022) are available here: 10.6073/pasta/2ebd7eaf2fac27e1c7eee9678baa7940.
