# Supplementary material for: Effects of elevated nutrient supply on litter decomposition are robust to impacts of mammalian herbivores across diverse grasslands
Source: Oecologia. 2025 Sep 13;207(10):157. doi: 10.1007/s00442-025-05791-4 (PMC12433350; doi:10.1007/s00442-025-05791-4)
Supplement: Supplementary file 1 — Supplementary file1 (DOCX 2071 KB) [file 442_2025_5791_MOESM1_ESM.docx]

**SI Materials**

*SI Table 1.* Litter decay parameters (mean +/- standard error) for each treatment at each site.

*SI Table 2.* Results from treatment only linear mixed models. *R*^2^_m_ (marginal *R*^2^) considers only the variance of the fixed effects, while *R*^2^_c_ (conditional *R*^2^) considers the variance of both the fixed and random effects. The standard deviation of the random effects intercept (RE StdDev) describes the magnitude of variation across sites.

*SI Table 3.* Descriptions of exceptions to the fence design; sites not included in this list have standard Nutrient Network design as described in Methods. Adapted from Borer et al. (2020).


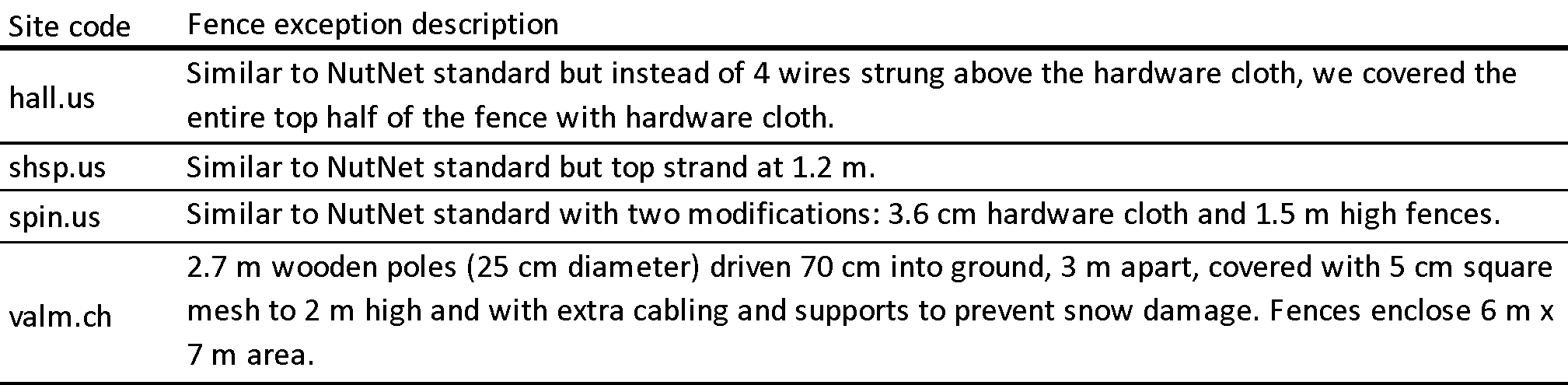

*SI Figure 1.* Treatment effects on aboveground live (a) and dead (b) biomass across all sites. Means and standard error bars shown for each treatment, with significant differences (P < 0.05) between treatments indicated with letters.

*SI Figure 2.* Herbivore intensity compared to the effect of herbivore presence on early-stage (t_1/10_) litter decay calculated as log response ratios. Each point represents a site, as identified by the site code, with dashed lines referencing no effect. There was not a significant relationship between herbivore intensity and the effect of herbivory on litter decay. A positive value for herbivore intensity indicates that the presence of herbivores stimulated aboveground biomass. A positive value for the herbivore effect on decay indicates that the presence of herbivores suppressed early-stage decay (i.e., increased t_1/10_).
